# Rapid volumetric photoacoustic tomographic imaging with a Fabry-Perot ultrasound sensor depicts peripheral arteries and microvascular vasomotor responses to thermal stimuli

**DOI:** 10.1007/s00330-017-5080-9

**Published:** 2017-10-10

**Authors:** Andrew A. Plumb, Nam Trung Huynh, Jamie Guggenheim, Edward Zhang, Paul Beard

**Affiliations:** 1 Centre for Medical Imaging, Division of Medicine, University College London, Podium Level 2, 235 Euston Road, London, NW1 2BU UK; 2 Photoacoustic Imaging Group, Department of Medical Physics and Biomedical Engineering, University College London, London, UK

**Keywords:** Photoacoustic techniques, Interferometry, Ultrasound, Peripheral vascular diseases, Vasoconstriction

## Abstract

**Purpose:**

To determine if a new photoacoustic imaging (PAI) system successfully depicts (1) peripheral arteries and (2) microvascular circulatory changes in response to thermal stimuli.

**Methods:**

Following ethical permission, 8 consenting subjects underwent PAI of the dorsalis pedis (DP) artery, and 13 completed PAI of the index fingertip. Finger images were obtained after immersion in warm (30-35 °C) or cold (10-15 °C) water to promote vasodilation or vasoconstriction. The PAI instrument used a Fabry-Perot interferometeric ultrasound sensor and a 30-Hz 750-nm pulsed excitation laser. Volumetric images were acquired through a 14 × 14 × 14-mm volume over 90 s. Images were evaluated subjectively and quantitatively to determine if PAI could depict cold-induced vasoconstriction. The full width at half maximum (FWHM) of resolvable vessels was measured.

**Results:**

Fingertip vessels were visible in all participants, with mean FWHM of 125 μm. Two radiologists used PAI to correctly identify vasoconstricted fingertip capillary beds with 100% accuracy (95% CI 77.2-100.0%, *p* < 0.001). The number of voxels exhibiting vascular signal was significantly smaller after cold water immersion (cold: 5263 voxels; warm: 363,470 voxels, *p* < 0.001). The DP artery was visible in 7/8 participants (87.5%).

**Conclusion:**

PAI achieves rapid, volumetric, high-resolution imaging of peripheral limb vessels and the microvasculature and is responsive to vasomotor changes induced by thermal stimuli.

***Key points*:**

• *Fabry-Perot interferometer-based photoacoustic imaging (PAI) generates volumetric, high-resolution images of the peripheral vasculature.*

• *The system reliably detects thermally induced peripheral vasoconstriction (100% correct identification rate, p < 0.001).*

• *Vessels measuring less than 100 μm in diameter can be depicted in vivo.*

**Electronic supplementary material:**

The online version of this article (10.1007/s00330-017-5080-9) contains supplementary material, which is available to authorized users.

## Introduction

Peripheral arterial disease (PAD) is a common, important condition, affecting approximately 27 million individuals across the USA and Europe [1]. Most patients with PAD have generalised atherosclerosis throughout the cardiovascular system, including significant coronary artery disease in one-third, meaning that cardiovascular deaths are increased six-fold [2]. Furthermore, severe PAD itself causes significant local morbidity via tissue necrosis and ultimately amputation or even death. Healthcare costs are substantial (>$4.5 billion annually in the USA alone), similar to cerebrovascular disease and cardiac failure [3]. Therefore, prompt identification and treatment of PAD are important to patients, healthcare services, and wider society [4].

PAD involves both large vessels (LVs) and small vessels (SVs) beyond named arterial branches. Whereas LVs are well depicted by existing techniques, primarily ultrasonography (US) and computed tomographic or magnetic resonance angiography (CTA/MRA), limited spatial resolution precludes meaningful assessment of SV. This is problematic, because SV-PAD is closely associated with diabetes mellitus [5], which is increasing in incidence globally [6]. Accordingly, there is clear need to develop newer techniques to accurately assess SV-PAD non-invasively.

One possible solution is photoacoustic (optoacoustic) imaging (PAI), which exploits the photoacoustic (PA) effect, whereby laser illumination of tissue provokes broadband ultrasound emission. These ultrasound waves can be reconstructed into high-resolution images, based on the optical absorption properties of the tissue [7]. PAI is particularly suitable for vascular imaging because haemoglobin has strong optical absorption, maximising ultrasound emission and image contrast. However, imaging at clinically relevant depths can be problematic because normal tissues have high optical and acoustic attenuation, mandating highly sensitive ultrasound detectors. Furthermore, it is challenging to simultaneously illuminate tissue and detect ultrasound without the ultrasound detectors obscuring the laser light. We have developed a PA system that uses a Fabry-Perot (FP) interferometer as the ultrasound sensor [8]. This is optically transparent to the excitation laser, avoiding such obscuration. Furthermore, the FP sensor outperforms conventionally used piezoelectric ultrasound sensors in sensitivity, bandwidth, and element size, yielding improving 3D image resolution and contrast [8] for sub-centimetre scale vascular imaging.

Several previous PAI studies have depicted human vasculature successfully, although most only imaged superficial skin vessels [9–11], or while investigating dermatological disease [12], although clear proof-of-concept of deeper vessel imaging has been reported, of both larger named arterial vessels [13] and within organs such as the breast [14]. A further recent article described high-resolution two-dimensional peripheral foot vessel imaging using a handheld probe and a concave ultrasound detector array [15]. Further ex vivo animal model work has shown that volumetric PAI can successfully depict subcutaneous veins during endovenous laser therapy [16]. However, PAI has not yet been shown capable of detecting vascular changes induced by either normal physiology or disease in humans, nor has it been subject to appropriately powered, prospective clinical studies with interpreter blinding and pre-specified endpoints aimed at clinical validation, as befits development of a new imaging technology [17]. Since SVs are readily able to vasoconstrict and vasodilate in response to cold and heat respectively [18], they are an excellent experimental test bed for evaluation of PAI-based vascular imaging, serving as a safe, laboratory-controlled paradigm for the constriction of the SVs seen in atherosclerotic disease. We therefore wished to determine if our FP sensor-based PAI system can successfully depict (1) peripheral leg arteries and (2) microvascular circulatory changes in response to thermal stimuli as a reliable, controllable means of safely inducing SV vasoconstriction and vasodilation.

## Materials and methods

Ethical permission was granted for this prospective single-centre study by the University College London Research Ethics Committee (Project ID: 1133/001).

### Participants

We recruited healthy volunteers, aged ≥ 18 years and taking no medication, via internal advertisement. Exclusion criteria were inability to provide informed written consent, vascular/cardiorespiratory disease, and any skin disorder preventing safe imaging probe placement. Ultimately, we recruited 15 volunteers (13 male); 13 participated in the thermal stimulus study and 8 in the limb artery imaging study (5 completed both studies).

### Imaging platform

**Fig. 1.**
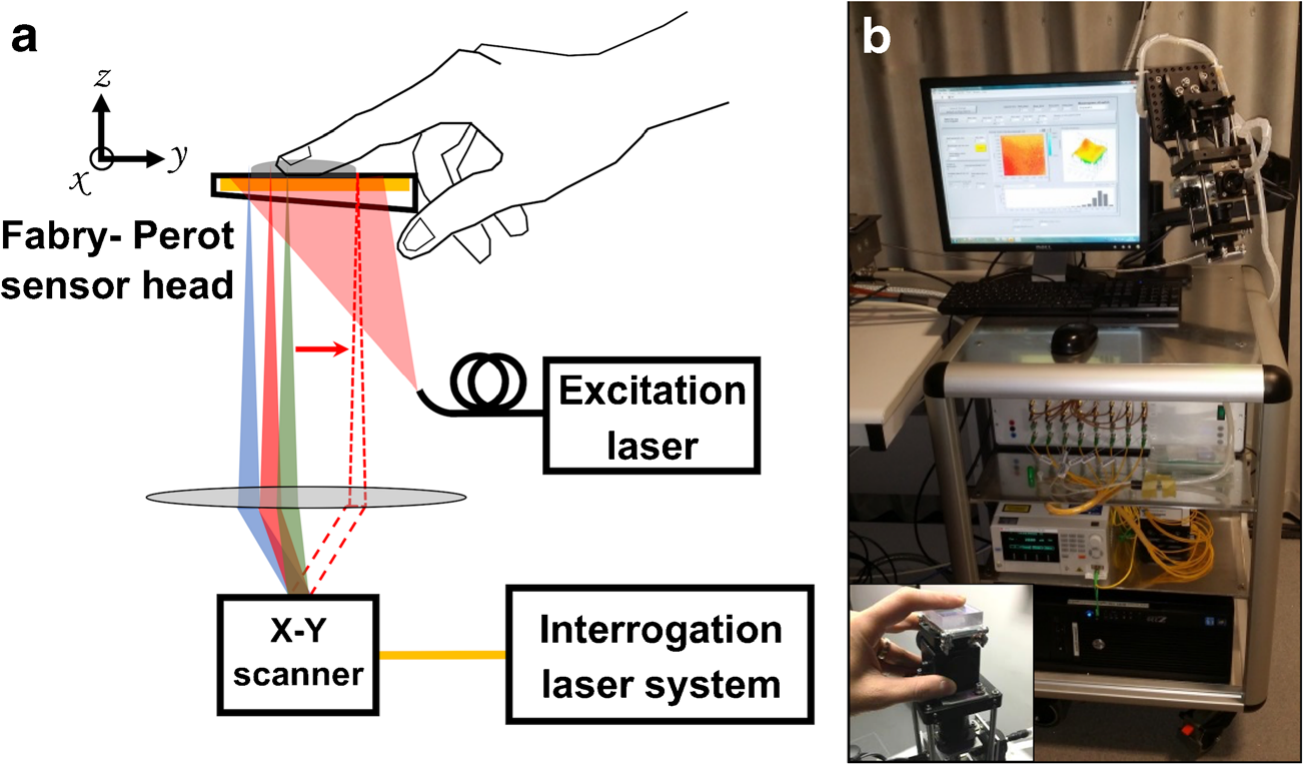
Fabry-Perot photoacoustic imaging system. A schematic of the imaging setup is shown in (**a**), with a photograph of the system being used for image acquisition (in this case, of the index fingertip) in (**b**), with detail in the *inset*

The PAI platform is based on a previously described prototype [19]. The system (Fig. 1) comprises a fibre-coupled “excitation” laser that illuminates the target tissue, provoking photoacoustic ultrasound emission for subsequent detection by the FP sensor. Here, we used a fibre-coupled, 30 Hz, optical parametric oscillator (OPO) excitation laser system (SpitLight-600, InnoLas Laser GmbH, Krailling, Germany) at a nominal wavelength of 750 nm. This wavelength balances high absorption by haemoglobin and good tissue depth penetration.

The FP interferometer comprises a polymer spacer sandwiched between two mirrors. Incident ultrasound waves modulate the spacer thickness, producing a corresponding change in optical reflectivity. This reflectivity change can be mapped very precisely in two dimensions by rapidly raster scanning the sensor with a second “interrogation” laser, thus synthesising a 2D array of tens of thousands of highly sensitive individual ultrasound detectors. We used a parallelised Santec TSL-550 1550-nm laser to interrogate the sensor and InGaAs photo-detectors (Hamamatsu G9801-22) to permit measurement of the optical power changes induced by the photoacoustic waves at a sampling rate of 60 MHz. The scanner provides a spatial resolution in the range 75-125 μm depending on the imaged depth and has an ultrasound bandwidth (-3dB) of 30 MHz. The field of view of the instrument is dependent on the area of the FP sensor that is scanned by the interrogation laser, typically 1-2 cm in each of the x and y dimensions. Depth of view (the z dimension) is governed by penetration of laser light into the imaged volume.

PAI reconstruction methods for an earlier scanner prototype have been described previously [8, 19, 20]. The algorithm uses a k-space fast Fourier transform method to reconstruct the photoacoustic image from the spatio-temporal distribution of the PA-induced ultrasound measured by the FP sensor. For immediate feedback at the time of scanning, rapid reconstruction was performed using non-upsampled data, generating images within 2-4 s. However, since the sensor captures higher frequency components in the time domain (60 MHz sampling rate) than in the spatial domain (scanning steps of 106 μm), it is possible to reconstruct higher-quality images by upsampling spatially (in this case, two fold) so that the high frequency information from the time domain is used in the reconstruction [21]. This increases the computation time to 10-20 s. Images were interpolated (×2) after reconstruction to generate voxels of approximately 25 μm for radiologist manipulation. DICOM images were exported for radiologist manipulation and interpretation (Osirix, Pixmeo Sarl, Switzerland).

### Imaging protocol

Images were acquired over two visits. On the first, participants submerged their non-dominant hand in a cold water bath (10-15 °C). After 3-min immersion, their contralateral (non-immersed) index fingertip was imaged to investigate for reflex vasoconstriction (“reflex stimulus”). At 5 min, the immersed hand’s index finger was removed from the water and immediately imaged (“direct stimulus”). Five minutes was chosen as the length of immersion because in normal physiology a more prolonged cold stimulus than this often induces vascular dilatation in order to protect the peripheries from tissue damage due to cold [18]. Images were obtained using a 20-mm-diameter excitation beam and incident fluence at the skin surface of 6.5 mJ, four fold below maximum regulatory exposure limits (BS-EN-60825-1). The sensor was scanned over a 14 × 14-mm area in 106-μm steps, thus acquiring 17,443 PA waveforms; acquisition time (for the entire imaged volume) was 90 s. Participants then transferred their non-dominant hand to warm water (30-35 °C) and the experiment was repeated for both the reflex stimulus (contralateral hand) and direct stimulus (immersed hand).

At a second visit, the dorsalis pedis (DP) artery was imaged. An experienced radiologist (A.A.P.) used the 40-MHz linear probe of a Viewsonics SonixMDP scanner (Analogic Ultrasound, Richmond, Canada) to obtain longitudinal and axial vessel images. Immediately thereafter, location-matched PAI images were obtained by positioning the PA scan head at the same position that had been used for sonography.

To determine if PAI could detect SV changes induced by thermal stimuli, we assessed the images (1) subjectively and (2) quantitatively. For the former, paired images for each participant in each imaging condition (i.e. cold and warm) were viewed independently in random order by two radiologists. Reflex and direct stimulus images were viewed separately. Each radiologist judged whether SVs were more readily visible after cold or warm water immersion. Subsequently, quantitative measures were obtained by using the DICOM viewer to count the number of voxels returning vascular signal. An elliptical region of interest (ROI) was drawn within the largest imaged vessel, and the vascular signal was defined as the mean signal intensity within this ROI, ± 2 standard deviations. The number of voxels within each imaged volume meeting these conditions was then measured. We also measured the dimension of PAI-depicted SVs by drawing a linear ROI across their short axis and calculating the full width at half maximum (FWHM).

DP artery conspicuity was judged subjectively on a four-point scale (0, imperceptible; 1, barely perceptible; 2, perceptible, but with some artefacts; 3, clearly visible, minimal/no artefacts) by viewing maximum-intensity projection (MIP) and multiplanar reformats (MPRs). For each artery, the depth from the skin surface to its superficial and deep margins was measured using MPR images.

### Power calculation

The primary power was based on readers’ ability to determine which of a given image pair had been obtained after cold water immersion (representing a vasoconstricted state) using the direct stimulus. We assumed that a correct identification rate of ≥ 90% (vs. the 50% expected by chance) would imply future clinical value. Therefore, at a power of 80% and significance level of 5%, we required 13 participants (G*Power version 3.1.9.2 for Mac).

### Statistical analysis

The primary outcome was the proportion of individuals in whom cold (vs. warm) water immersion was correctly identified by the radiologists for the direct stimulus. This was calculated separately for each reader and compared to chance (50% rate) using a one-sample test of proportions. Secondary outcomes were the results of the qualitative radiologist assessment for the reflex stimulus and the number of voxels demonstrating vascular signal (for the direct and the reflex stimulus). The latter were compared for cold vs. warm images using the Wilcoxon signed-rank test. Descriptive statistics were calculated for other outcomes. We used R version 3.2.0 for Mac software, taking probability values of < 0.05 as significant.

## Results

There were no symptomatic side effects from PAI for any participant. No skin damage, irritation, or discomfort was reported either immediately following image acquisition or in the days thereafter. High-quality, three-dimensional spatially resolved data sets were successfully acquired in all cases. Average acquisition time was 90 s; the SV experiment was completed in approximately 15 min (including the 10 min of water immersion time, half for each hand) and the LV experiment was completed in approximately 5 min.

### Fingertip SVs

#### Subjective assessments

**Fig. 2.**
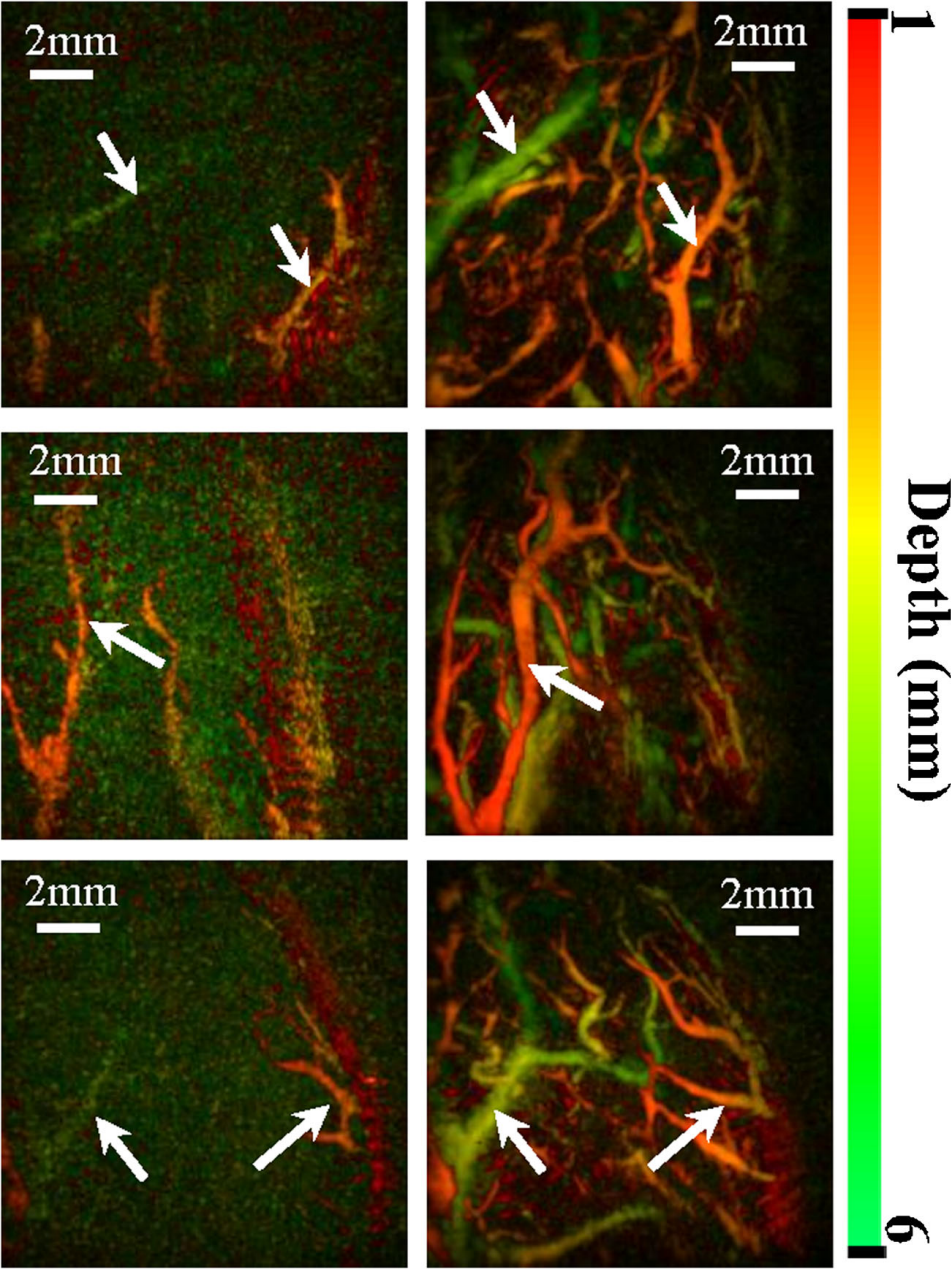
Maximum intensity projection (MIP) fingertip PA images after cold (*left hand panels*) and warm (*right hand panels*) water immersion using the direct stimulus, in three different subjects, color-coded for depth. *Arrows* show the same vessels in each imaging condition

SVs were depicted successfully in all 13 volunteers. Both readers judged there to be fewer visible vessels after cold water immersion vs. warm in all cases for the direct stimulus (correct identification rate = 100%, 95% CI 77.2 to 100.0%, *p* < 0.001, Fig. 2 and Supplemental Material 1 and 2). For the reflex stimulus, both readers judged there to be fewer visible SVs after cold water immersion in all cases except one (in which the two conditions were judged equivalent; correct identification rate = 92.3%, 95%CI 66.7 to 98.6%, *p* = 0.006).

#### Quantitative measurements

**Fig. 3.**
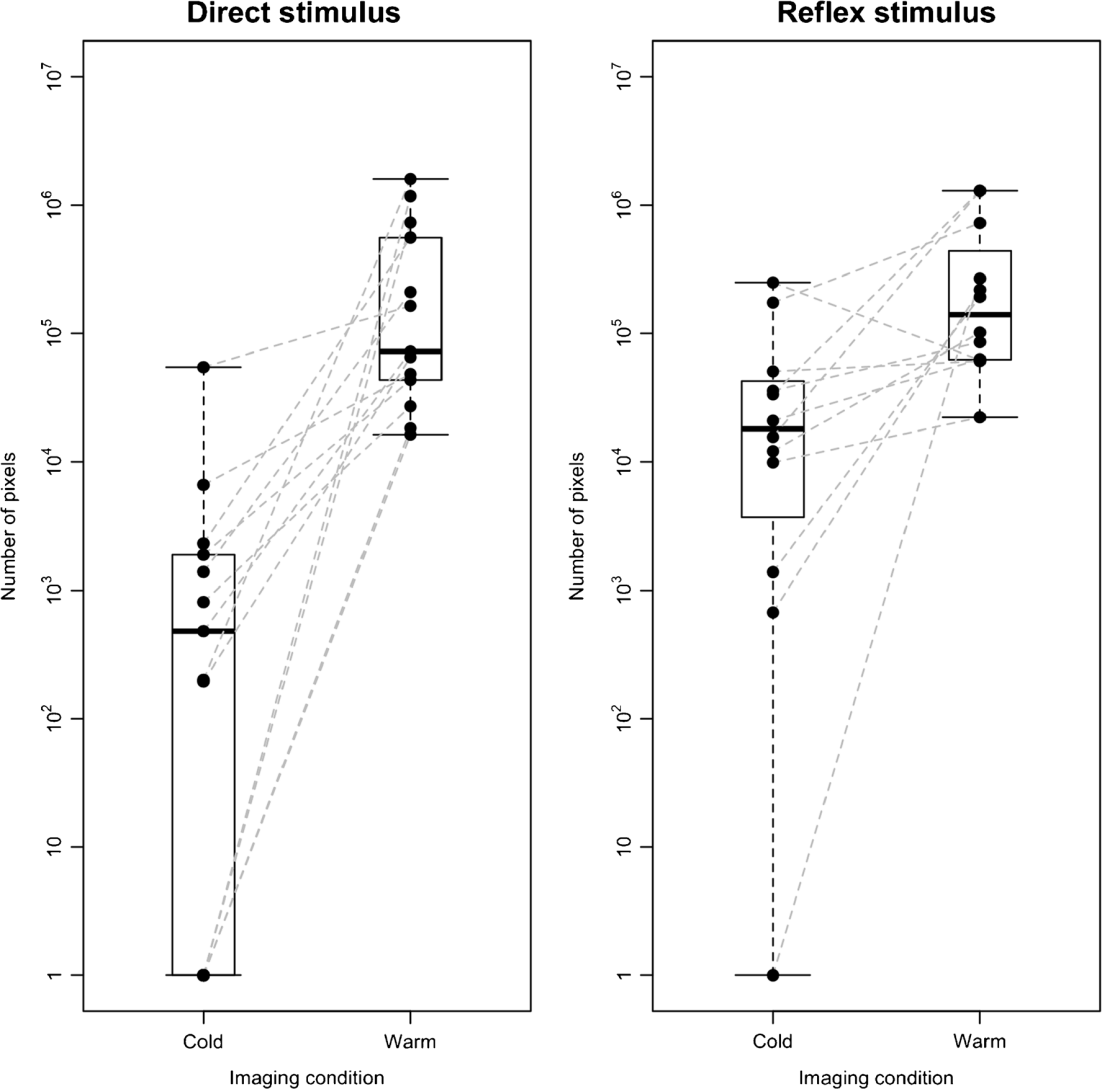
Boxplots and strip charts showing the number of voxels exhibiting vascular signal (y-axis, logarithmic scale) for all subjects in each imaging condition (x-axis). Dashed grey lines show the change for each individual subject. Panel (**a**) shows results for the direct thermal stimulus, in which all subjects showed an increase in the number of vascular signal voxels after immersion in warm vs. cold water. Panel (**b**) shows results for the reflex stimulus: All subjects except one showed an increase after warm water immersion of the contralateral hand

The mean number of voxels exhibiting vascular signal was significantly lower after cold water immersion than warm water immersion for both the direct stimulus and the reflex stimulus (direct: cold = 5263 voxels; warm = 363,470 voxels, *p* < 0.001; reflex: cold = 50,388 voxels, warm = 365,037 voxels, *p* = 0.007). Each individual volunteer showed less PAI signal after cold vs. warm water immersion after the direct stimulus (Fig. 3a), mirroring the 100% discrimination recorded subjectively. For the reflex stimulus, only a single subject had more PAI signal after cold vs. warm water (Fig. 3b). The smallest vessels depicted by PAI had mean FWHM dimensions of 125 μm (range: 75–150 μm). These tiny vessels were completely undetectable by 40-MHz B-mode and Doppler ultrasound (not shown).

### Peripheral limb arteries

**Fig. 4.**
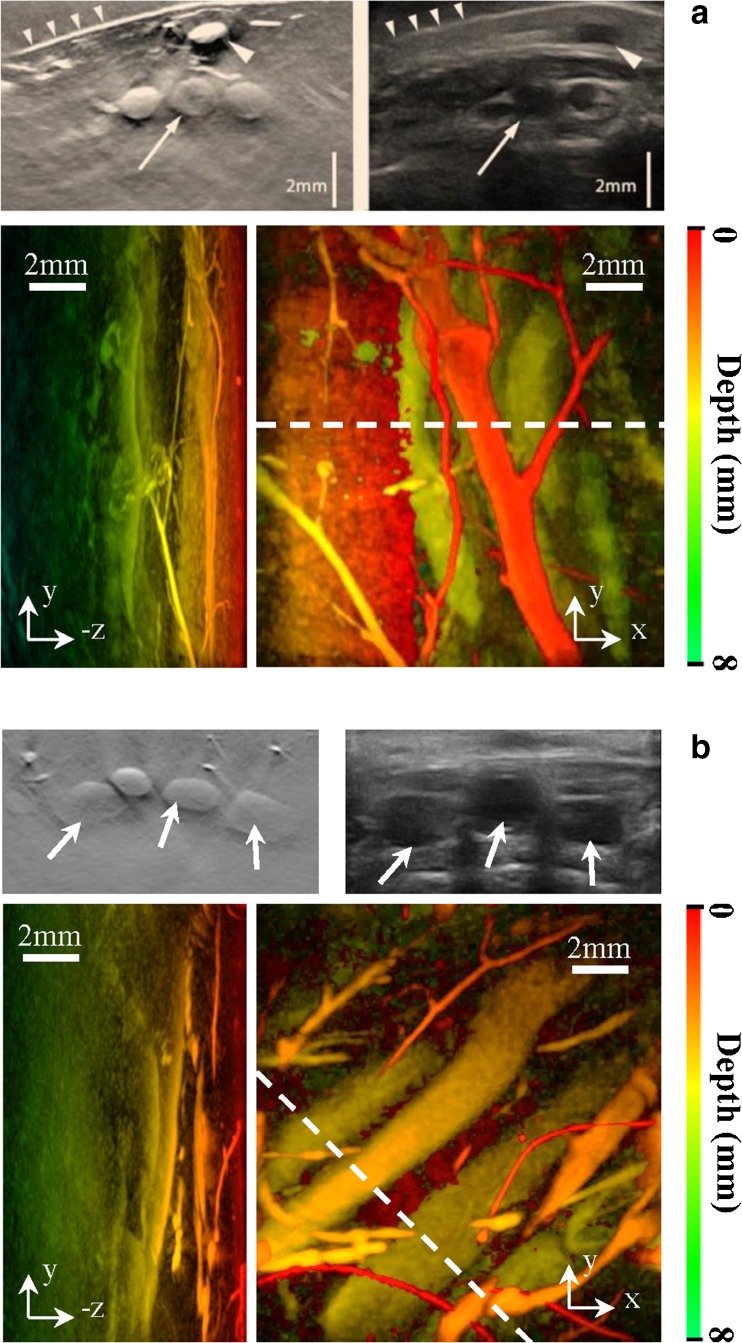
Example short axis 2D slices taken from the volumetric PA imaging series (*left upper panels*) with contemporaneous, location-matched ultrasound (*right upper panels*) for two separate volunteers (**a** and **b** respectively). The corresponding PAI volumetric data sets, presented as coronal and sagittal maximum intensity projection (MIP) images, are shown in the *lower panels*. MIP images are color-coded for depth (see scale). The *dashed line* indicates the plane through which the axial image has been reconstructed. The DP artery, with its accompanying venae comitantes, is arrowed, with a superficial vein (*large arrowhead)* and the skin surface (*small arrowheads*) also shown in **a**. The axes are as follows; y, proximal-distal; x, medial-lateral; z, dorsal-plantar

**Fig. 5.**
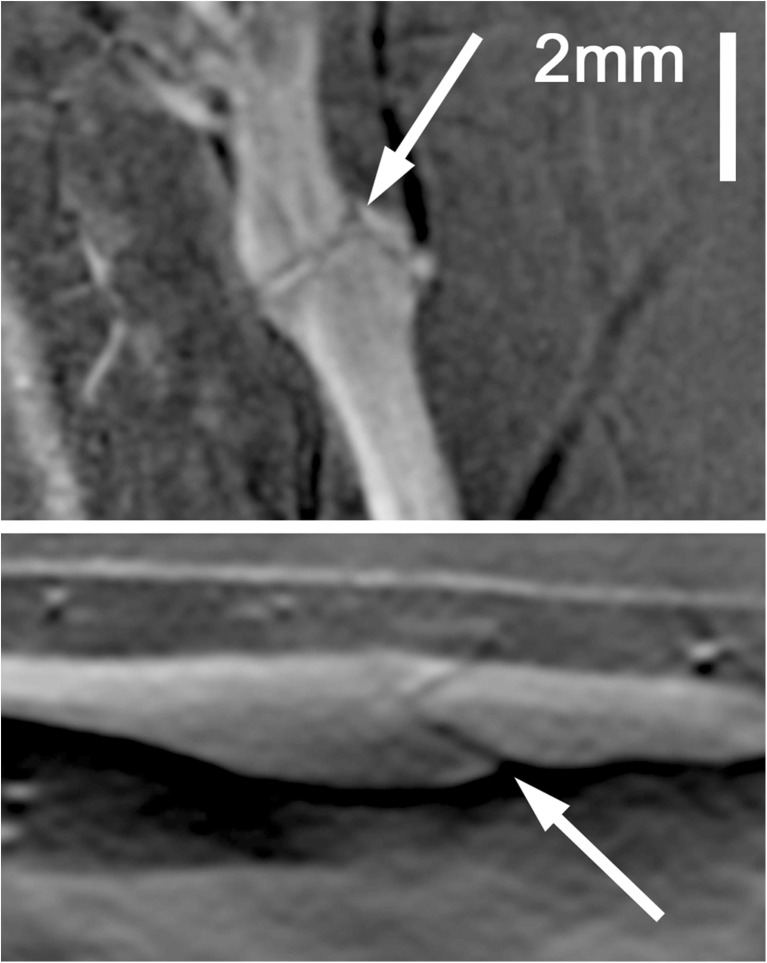
Example coronal and sagittal maximum intensity projection (MIP) images through a superficial foot vein of a healthy volunteer, depicting a pair of angled, linear webs of reduced signal (*arrows*) within the vessel at a site of slight venous dilatation. These were not visible on ultrasound, but from their morphology and location they are presumed to represent a venous valve

Images were reconstructed to depths of 14 mm. The dorsalis pedis (DP) artery was visible (score of ≥ 2) for all eight participants except one (examples in Fig. 4 and Supplementary material 3). This participant had dark skin (Fitzpatrick scale = 6) and deeper vessels were obscured by strong laser absorption by melanin at the wavelength used. A subsequent attempt at re-imaging this individual at a different excitation wavelength (900 nm) was successful. Mean distance from the skin to the DP was 2.9 mm to its superficial border (range: 1.8 to 4.5 mm) and 4.6 mm to its deep border (range: 3.5 to 6.9 mm). Several accompanying veins demonstrated internal fold-like structures, taken to be normal venous valves (Fig. 5).

## Discussion

We have shown that PAI generates high-resolution, three-dimensional images of both SV and LV *in vivo* and depicts thermally induced peripheral vasoconstriction, both directly and via reflex action. Importantly, this was using a robust experimental design with pre-specified outcomes, adequate sample size to achieve statistical power, and radiologist blinding during image interpretation. The system has a convenient, easily manipulated probe and acquires volumetric images rapidly (< 90 s to acquire over 17,000 PA waveforms and image at 14 × 14 × 14-mm volume). The findings suggest that PAI may be a powerful technique for assessing arterial disease.

Our PA system uses a different method of ultrasound detection from most previously described devices, namely, a Fabry-Perot interferometer (vs. a piezoelectric array). This has several major advantages; first, it can be placed directly on tissues of interest without obscuring the excitation laser, simplifying imaging geometry. Second, FP sensors are highly sensitive to ultrasound. Third, it is possible to parallelise sensor interrogation by using a multi-beam laser, thereby accelerating image acquisition considerably. Here, we accelerated eight fold; future upgrades to 16- and 24-beam systems are planned and will reduce acquisition times further (analogous to multi- vs. single-detector row CT). Theoretically, such parallelisation is limited solely by the technical complexity and cost, although in practical terms, scanning more than 100 channels will be challenging. As well as these hardware improvements, it is possible to interrogate the FP sensor using compressed sensing techniques, which speeds acquisition by reducing the amount of data that needs to be collected without significantly compromising image quality [22]. As well as these approaches on the “sensor read-out” side of the device, the excitation step can also be hastened by using excitation lasers with a higher pulse repetition frequency. Lasers similar to that used in the current study but operating at 200 Hz (vs. 30 Hz) are commercially available and would reduce the acquisition time to <30 s per imaged volume. Even faster rates are possible using alternative technology such as fibre lasers [23]. However, ultimately this will be limited by more rapid energy deposition in the imaged volume, potentially contravening safety limits. We anticipate that up to 1 kHz excitation lasers could, in theory, be deployed successfully and safely (using energies of a few mJ). The combination of all these elements means that video frame-rate three-dimensional acquisition is ultimately likely to be achievable. Fourth, the system is ideally suited to refinement by adding conventional ultrasound (for detection by the same FP sensor), which will permit fused, perfectly co-registered US/PAI images from a single device. Finally, the sensor can be constructed in a variety of geometries, permitting adaptation to specific clinical tasks (e.g. laparoscope- or endoscope-mounted sensors for intra-operative or endocavitary imaging [24]).

A concern for the clinical application of PAI is its relatively limited penetration depth [25]. This problem arises primarily because of optical attenuation (i.e. inability of the laser light to reach the imaging target) and, to a lesser degree, because of sonographic attenuation (i.e. inability of the laser-generated ultrasound to reach the detector). Despite these concerns, the data reported here show that we were able to routinely depict the DP artery at depths of several millimetres from the skin surface, meaning larger vessels are well within reach of the instrument. Accordingly, PAI as employed here is highly complementary to conventional Duplex US, which images larger and deeper vessels effectively. Presently, PAI is unlikely to compete directly with US for macrovascular imaging, but instead permits high-resolution, volumetric imaging of the SVs that Duplex US cannot detect. Nonetheless, we anticipate that PAI imaging depths will increase further. For example, it will be possible to increase the power of the incident excitation laser since we imaged at powers well below the maximum tolerable limit. Additionally, more sensitive ultrasound detectors are being developed [26] and will likely improve imaging depths from the current maximum of around 1 cm (using the present system) to over 2 cm. Other groups have also successfully used PAI based on a concave piezoelectric ultrasound array to depict both LVs and SVs with similar resolution to that reported here [15]. These highly complementary data to our own strongly imply that the underlying PAI technology will translate successfully to routine clinical practice.

There are several limitations to our study. First, we recruited healthy volunteers and induced vasomotor changes using thermal stimuli, which may not be representative of changes in disease. It is possible that the degree of vasoconstriction induced by cold water immersion is greater than that caused by atherosclerosis of typical severity. These data should therefore be taken as proof of the concept that PAI can depict changes to vascular beds in general rather than atherosclerosis specifically. Second, we imaged only at a single wavelength; since haemoglobin and deoxyhaemoglobin have different absorption spectra, it is theoretically possible to estimate oxygen saturation by multi-wavelength imaging [27], an important future avenue for development. Third, we imaged volumetrically in 3D mode, sacrificing temporal resolution and real-time device navigation for a larger field of view. The device is entirely capable of real-time 2D imaging (at frame rates exceeding 10 frames/second) and in the future we plan to permit user-controlled switching between the two, 2D for device navigation and positioning and 3D for detailed assessment. Finally, for reasons of convenience and practicality we imaged volunteers’ fingertips rather than their toes, which is the more common location for peripheral arterial disease—it is possible (although unlikely) that vasomotor changes differ between these sites.

In summary, photoacoustic imaging using a Fabry-Perot interferometer-based device successfully generates three-dimensional images of human vasculature and permits detection of vasomotor microcirculatory changes induced by thermal stimuli. These data suggest it may have value in the evaluation of patients with arterial disease.

## Electronic supplementary material

ESM 1 (MOV 22398 kb)

ESM 2 (MOV 21704 kb)

ESM 3 (MP4 8124 kb)

## Abbreviations

DP: Dorsalis pedis
FP: Fabry-Perot
FWHM: Full width at half maximum
LV: Large vessel
PA: Photoacoustic
PAD: Peripheral arterial disease
PAI: Photoacoustic imaging
PAT: Photoacoustic tomography
SV: Small vessel
SVD: Small vessel disease
